# Cognitive Regulation Strategies Used by Children with Reading Difficulties

**DOI:** 10.3390/children11030288

**Published:** 2024-02-28

**Authors:** Carmen David, Cristina Costescu, Alina Frandeș, Adrian Roșan

**Affiliations:** Special Education Department, Faculty of Psychology and Educational Sciences, Babe-Bolyai University, 400029 Cluj Napoca, Romania; carmen.david@ubbcluj.ro (C.D.); alina.frandes@stud.ubbcluj.ro (A.F.); adrian.rosan@ubbcluj.ro (A.R.)

**Keywords:** reading comprehension, emotion regulation, off-task thoughts

## Abstract

(1) Background: Children with reading difficulties may experience negative emotions and social isolation. The cognitive emotion regulation strategies that they use in different reading tasks can make them more vulnerable in the stressful situations. Using adaptative emotion regulation strategies may help them overcome stressful reading situations. (2) Methods: Children identified with poor reading comprehension skills were compared to children without reading comprehension difficulties on measures of self-evaluation and thought in relation to task, and on cognitive coping strategies relevant to performance. The effect of some relevant demographic factors was also investigated, such as gender and urban/rural setting. (3) Results: Our results indicate that children that have poor reading comprehension skills present higher scores on negative self- evaluation and off-task thoughts that are in relation to performance anxiety. Also, in what concerns cognitive coping strategies, students with difficulties in reading comprehension display a greater use of blaming others strategy, which is in relation to the negative self-evaluations. Also, they displayed less use of putting into perspective. No effect of gender and setting emerged for off and on task thoughts and cognitive coping strategies, except for lower scores of students from rural setting in positive self-evaluation. (4) Conclusions: students presenting difficulties in reading comprehension tend to use more negative self-statements and disengagement through off-task thoughts and employ coping strategies directed to protect self-worth.

## 1. Introduction

There is an increased recognition of the emotional impact that the ability to regulate emotions has on the learning process [1]. The ability to regulate emotions through cognitive processes or thoughts empowers adolescents to control their emotions and orient their actions toward achieving their goals even when facing stressful or challenging events [2,3]. According to Gross [4], emotional regulation refers to external or internal ways of evaluating, analyzing, and altering emotional responses, more specifically, the attempts that individuals make in order to influence how their emotions are experienced and expressed. Coping is also linked to academic performance in adolescents, which refers to one’s efforts to manage the relation between the environment and the ability of the individual to respond to the challenges [5]. The two constructs, coping and emotional regulation, may overlap; however, coping includes actions that are not necessarily linked with emotion that are taken by an individual to achieve their goal, whereas emotional regulation is linked with emotions, and the two constructs appear in different contexts. In the emotional regulation model proposed by Gross, the most investigated emotional regulation strategies that involve cognitive changes are cognitive reappraisal and expressive suppression [6]. Cognitive reappraisal is an antecedent-focused strategy and refers to a strategy where one reinterprets the meaning of situations to modify their own emotional response [7]. On the other hand, expression suppression refers to the inhibition of the ongoing emotion-expressive behavior, without modifying the emotional experience [8]. 

The regulation of emotions through cognitions or thoughts is associated with the capacity to manage one’s emotions after facing stressful events [9]. Even though emotional regulation is a universal capacity, there are specific differences in thoughts or cognitions that people use. Garnefski and collaborators [10] suggested that there are more than two important cognitive emotional regulation strategies, and they also associate some of the strategies with vulnerability to emotional problems. The nine conceptually different emotional regulation strategies that encompass both adaptative and maladaptive strategies are self-blame, acceptance, rumination, positive refocusing, refocus on planning, positive reappraisal, putting things in perspective, catastrophizing, and blaming others. Several studies have investigated and proved the relationship between rumination, self-blame, and catastrophizing and emotional problems in adolescents [11,12]. 

School-related stress can lead to negative emotional development in children and adolescents, including anxiety and shame patterns or anger and frustration [13,14]. Adolescents who experience emotional or behavioral problems are at risk of future negative outcomes, such as dropping out of school, social isolation, suicide ideation, and developing affective disorders [15,16]. Among the most prevalent school related-stress is reading difficulties. Children with persisting reading difficulties show elevated emotional and behavioral problems and are more likely to experience symptoms of depression or anxiety compared to good readers [17,18]. Morgan and his collaborators [19] investigated the predictive association between 8-year-old children’s reading ability and 10-year-old children’s social and emotional outcomes, and they found that poor reading abilities make children more likely to exhibit emotional symptoms like sadness, anger, or social isolation compared to good readers. Classroom activities that involve reading can be considered stressful for poor readers also because they are not controllable or chosen by children. Therefore, children and adolescents with reading difficulties are more prone to use cognitive emotional regulation strategies than coping mechanisms, considering the fact that they have little control over the stressful event (a reading activity) [20]. One strategy that can be also used by children is disengagement, which represents a form of withdrawing from the stressor and involves reducing the effort toward the task, which may lead to less engagement in more intensive practice [21]. Disengagement and off-task thoughts are hypothesized to impair one’s performance, not only on reading tasks. Hollandsworth and his collaborators [22] suggested four types of cognitions that might influence the performance of anxious individuals in a testing situation: positive and negative self-evaluation and on- and off-tasks thoughts. Whereas low-test-anxious people are more prone to engage in positive self-evaluation and on-task thoughts, people who have high test anxiety are more likely to have off-task thoughts and negative self-evaluation.

Limited research has investigated the emotional regulation strategies or affective experiences of children and adolescents with learning difficulties. Children with learning disabilities, in addition to their low achievement, have difficulties in socializing, which is linked to isolation and anxiety [23,24]. Moreover, the results from a meta-analysis revealed that the prevalence of depression among students with learning disabilities was estimated to be 88% of the reviewed studies [25]. The role of gender in the relation between learning difficulties and anxiety was investigated previously; however, mixed results were reported. Even though several studies revealed findings showing that girls report higher levels of anxiety than boys [26], Barnes and her collaborators [27] did not find evidence that gender moderated the relation between anxiety and reading comprehension. Despite the importance of reading comprehension for the learning process, on the one hand, and the influence of emotional regulation strategies on the other hand, apparently, there are no studies focusing on the relationship between the two, considering also adolescents’ beliefs about the cognitive strategies used. The relation between emotions and reading comprehension is bidirectional; for example, Bohn-Gettler and Rapp [28,29] demonstrated that some particular moods (happy and sad) can influence reading comprehension and post-reading memory. On the other hand, a recent study [30] investigated whether literacy difficulties in second and third grade were associated with higher levels of social and generalized anxiety. More than 100 children were examined, and their results showed that children with both reading and spelling difficulties had increased levels of anxiety compared to children with typical literacy development. Similar results were reported in a bigger study that involved 536 students with reading difficulties [31] that investigated if the dimensions of anxiety are related to reading comprehension, word reading fluency, and text reading fluency. 

The purpose of this study was to expand our understanding and knowledge on the stress response mechanism in relation to low school performance. To achieve this, we addressed the differences in cognitions in the school context and in coping strategies between children with poor and good reading comprehension skills. We decided to focus on text comprehension difficulties, because the extraction of meaning from texts represents the ultimate goal of the reading process. Successful reading comprehension requires language and reading processing abilities, such as decoding words, reading fluently, understanding the meaning of the words (vocabulary), linking the text with prior knowledge, and monitoring text understanding [32].

Our research questions are as follows: a. Are there differences in cognitions in the school context between those with poor reading comprehension skills and those without? b. Are there differences in the coping strategies of children between those with poor reading comprehension skills and those without? c. Are there gender differences in the coping strategies employed by children with poor reading comprehension skills? d. Are there gender differences in the cognitions in the school context of children that have reading difficulties? e. Are there differences in the cognitions in the school context between children with poor reading comprehension skills from rural schools and those in urban settings? f. Are there differences in the coping strategies employed by children with poor reading comprehension skills from rural schools or those in an urban setting?

## 2. Materials and Methods

### 2.1. Participants 

A total of 167 fifth graders participated in this study, after obtaining informed consent from their parents. Of the 167 students, 81 were females and 86 were males, all aged 11 to 12 years old. A total of 69% of the students were from urban schools, while 31% were from rural schools, but close to a major city (see Table 1). Children were attending three different mainstream schools. Schools were informed about the study and agreed. Afterwards, an informed consent form was sent to the parents. Children were also informed about the project. Only those who received informed consent from their parents were included in the study. Participants were categorized into a poor comprehension group or a typical comprehension group based on their MT-2 reading comprehension score. The cut-off point for selection was a raw score of 5. A raw score of 5 or less, on the test, indicates the student needs monitoring (a raw score of 5) or immediate intervention (scores from 0–4). A total of 28 students out of the 167 scored below 6. Students were not attending any support program. They followed the regular curriculum for 5th grade.

### 2.2. Instruments 

The data were collected in November 2022–January 2023. Firstly, we obtained the school management’s agreement and the informed consent from the parents. Afterwards, we proceeded with the administration of the reading performance measures, the Children Cognitive Assessment Questionnaire and the Cognitive Emotion Regulation Questionnaire. The reading fluency task was administered individually. The rest of the instruments were administered collectively.

Reading performance was measured by the Romanian version of MT-2 [33]. We employed a standardized comprehension task for fifth grade students and a standardized text fluency task for fifth grade students. The standardized tasks were adapted in Romanian [33] based on the MT-2 Italian version. The comprehension task consisted of silently reading a text and answering 10 comprehension questions. The maximum score was 10. Reading speed was measured via a score of syllables/second. 

The Children Cognitive Assessment Questionnaire (CCAQ) [34] is a self-administered instrument that measures cognitions in relation to test anxiety and task performance. It has 40 items, with 10 items per scale. It measures positive (PSE) and negative self-evaluations (NSE), self-distracting thoughts (off-task) (OFFT), and task-focusing thoughts (on-task) (ONT). Answers are dichotomist (true or false). Total score per scale is represented by the number of items for which the answer is applicable to the respondent. [32] reports the alpha Cronbach internal consistency coefficients per scale (NES = 0.82; PSE = 0.74; ONT = 0.67; OFFT = 0.72). We opted for an instrument that allows us to assess cognitions of self-evaluation and thoughts that can support/distract productive activities in the class, both being related to poor school performance. Poor performance has an impact on perceived competence and self-efficacy, as well as on self-evaluation. 

The Cognitive Emotion Regulation Questionnaire (CERQ) [35] measures cognitive coping strategies in response to stressful events through 9 scales: self-blame, other-blame, rumination, catastrophizing, putting into perspective, positive refocusing, positive reappraisal, acceptance, and refocus on planning. In [36], the authors report the internal consistency coefficients per scale, calculated on an adult sample. The coefficients range from 0.75 for self-blame to 0.86 for refocus on planning.

## 3. Results

The descriptive statistics (means and standard deviations) for the scores of the CCAQ of the two groups are presented in Table 2.

The differences between groups are small for two of the subscales. To address the statistical significance of the differences, we used an independent sample t test. There was no significant difference between groups for positive self- evaluation (*t* (35.04) = 1.75, *p* = 0.089) and for on-task thoughts (*t* (39.65) = 0.276, *p* > 0.05). A significant difference emerged, however, for negative self-evaluation (*t* (165) = −2.346, *p* < 0.05) and off-task thoughts (*t* (34.442) = −2.395, *p* < 0.05). 

The descriptive statistics (means and standard deviations) for the scores of the CERQ of the two groups are presented in Table 3.

To address the effect of reading comprehension difficulty on cognitive coping strategies, we used an independent sample *t*-test. The results are presented in Table 4.

Significant differences were obtained for two cognitive strategies, other-blame and putting into perspective, such that students in the poor comprehension group were less likely to use the strategy of putting into perspective and more likely to use the strategy of blaming others. 

In order to investigate the effect of gender on cognitions in relation to performance in children that have poor reading comprehension skills, we used a nonparametric test, the Mann–Whitney test. No significant difference was observed based on our data. Also, an identical procedure was used to analyze the effect of gender on cognitive coping strategies. No significant differences were obtained. 

We also tested for the effect of setting, such as urban or rural. No effect was evidenced for cognitive coping strategies. However, a significant effect was observed for positive self-assessment, such that students from a rural setting exhibited less positive self-statements than students from an urban environment. No other differences were significant on the CCAQ measure.

## 4. Discussion

The main aim of our study was to identify the differences in cognition in the school context of children aged between 11 and 12 years old that may exist between those with poor reading comprehension skills and those without. Moreover, our goal was also to investigate the differences in the coping strategies between children with poor reading comprehension skills compared with children without comprehension difficulties, as well as the role of gender or whether they are from urban or rural settings. Our findings suggest that children experiencing challenges in reading comprehension tend to exhibit higher scores of negative self-evaluation and off-task thoughts, which are related to performance anxiety. Furthermore, children with reading comprehension difficulties employ the other-blame approach more, which is linked to negative self-evaluation, while using less adaptative regulation strategies, such as putting things into perspective. Gender and setting did not show significant effects on the cognitive strategies used by the children, except for a lower positive self-evaluation score observed in students from rural settings. 

Children that have poor reading skills experience stressful situations in school settings. Moreover, children in fifth grade, while transitioning from elementary to middle school, are required to adjust to new teachers with different styles, new classroom colleagues, and new evaluative contexts in terms of academic performance. In the current study, we investigated differences in cognitions in the school context and in coping strategies between children with poor and good reading comprehension skills. 

Our results indicate that children that have difficulties in reading comprehension report more elevated scores on negative self-statements and off-task thoughts than children without such difficulties. This finding is important, as negative self-evaluations impact self-worth. However, ref. [37] found evidence that specific negative self-perceptions (related to low competence in reading) do not impact global self-worth. The authors explain this by the intervention of protective factors such as family support, teacher and peer support, or emphasis on areas of ability. However, a lack of a formal learning disorder diagnosis may contribute to less support and understanding from parents and teachers [38]. In our study, students presented reading difficulties, but they did not receive a formal diagnosis. Refs. [39,40] indicated that negative self-evaluation and off-task thoughts are more associated with test anxiety. Hollandsworth, in his study, ref. [41], indicated self-evaluation and thoughts related to the task as important for test anxiety. In a recent meta-analysis, ref. [42] revealed that there is a moderate risk for anxiety problems in individuals that have poor reading skills. However, while analyzing the moderator effect of reading subtype, they did not identify studies addressing only poor comprehension. 

In our study, off-task thoughts in children with poor comprehension skills are more present, compared to children that have good reading comprehension skills. For all children, reading tasks are difficult and require effort, and for many children, reading tasks are uncomfortable and activate thoughts that are debilitating overall for performance. On the other hand, these off-task thoughts may have a coping function or may be precursors to the emotional regulation strategy of disengagement. Such thoughts are considered debilitating to performance, as they interfere with cognitive engagement in a task and contribute to off-task behavior or task avoidance, which further contributes negatively to developing or improving a certain skill. Positive self-evaluation did not differ significantly between children without comprehension difficulties and those with. 

When investigating the cognitive coping strategies that are used by children with poor comprehension skills, the difference resided in higher scores on blaming others and lower scores for putting things into perspective. Blaming others is a coping strategy employed by students that encounter difficulties in reading. In order to overcome feelings of guilt, they may employ such a strategy. This strategy is more related to stressful events of relational experiences [3]. Paris and his collaborators [43] previously indicated that individuals with learning disorders develop counterproductive coping strategies. The other- blame strategy is considered a maladaptive strategy in which the individuals blame the context or other people for the negative events that they are experiencing. Some studies highlight this strategy as a predictor of stress [44]. This result is also consistent with evidence on attributional style in children with reading difficulties. In the event of failure, individuals with a learning disorder attribute the cause to subject or test difficulty [45]. 

Putting into perspective is a coping strategy through which the individual reduces the importance of an event or compares it to other situations to reduce its importance. In our study, children with reading comprehension difficulties use this strategy less. Our results differ from [46], in the case of children with dyslexia. Children with dyslexia employed this strategy with the aim of protecting self-esteem. However, this coping strategy was either adaptive, such as in the form of self-talk to assure persistence in difficult situations or in the form of referencing cases of dyslexia involving a normal life, or maladaptive, such as using self-talk to decrease the importance of standards [46,47].

From our knowledge, no previous study has investigated the differences between children with or without comprehension difficulties in terms of their cognitive emotion regulation strategies. However, late-identified children with reading difficulties have been reported to use the disengagement coping style more, which can be associated with off-task thoughts [21]. 

We investigated a gender effect on self-evaluation and thoughts related to the task. No significant differences emerged. Students with poor reading comprehension skills employed more negative self-evaluations. When addressing gender differences in coping strategies, a similar result was obtained based on our data, even though we expected differences. Alexander-Passe [48] reported differences between males and females in employing coping strategies, such that males used more task-oriented strategies, while females used more emotional regulation strategies and avoidance. The instrument we used in our study, however, measured cognitive coping strategies. 

Concerning the school setting, particularly the urban/ rural dimension impact on self-evaluation and debilitating or facilitative thoughts in relation to task performance, we did not find notable differences on negative self-evaluations and debilitating and facilitative thoughts, except for positive self-evaluations. Children from rural settings obtained lower scores on the positive self-evaluation subscale compared to children from urban settings. This result differs from [49]. However, they addressed more general self-worth and self-esteem in children not identified with reading comprehension difficulties. In our study, no differences emerged for cognitive strategy styles used, as measured by the CERQ. This result is consistent with [50]. They did not find notable differences in stressors and coping strategies on this dimension.

## 5. Practical Implications

An important finding was that children with difficulties in reading comprehension experience higher levels of negative self-evaluation and off-task thoughts, which are related to performance anxiety. This result emphasizes the importance of designing and implementing multicomponent interventions in cases of reading difficulties that include a component addressing the social—emotional factors in relation to reading performance, including effective coping strategies in the case of reading difficulties. Specialists working with children with reading difficulties should address the self- evaluation component through cognitive restructuring, self-acceptance, mindfulness, and the provision of constructive feedback [19,51] that emphasizes in successful performance use of the correct strategy, effort, perseverance, and reassurance of an adequate level of ability to pursue a task. Also, counseling sessions with parents are recommended in order to provide them with cognitive techniques to offer feedback, respond to low achievement, and motivate children to read. Moreover, since negative self-evaluation may be related to feelings of guilt, the screening and identification of children that experience difficulties in reading comprehension may relieve them of such negative feelings and may lead to more positive self-evaluation [37,50] based on this newly acquired information on their condition (such as a learning disorder). Therefore, when persistent difficulties in reading comprehension are observed, the classroom teacher should make the recommendation for further evaluation. Since negative self-evaluations are also linked to attributional styles, ref. [51,52] suggests attribution retraining and adequate feedback provision. 

## 6. Limits

Our study emphasizes the importance of addressing emotional factors in the case of reading difficulties; however, it has some limitations. Firstly, participants were not formally diagnosed, and we only used one measure, though adequate, for the selection process. Therefore, our results should be interpreted with caution, because we do not know if our findings can be generalized to children with learning disabilities. Another limitation concerns the fact that we only included in our investigation some cognitive coping strategies, while not investigating other types of coping strategies in relation to reading difficulties. Moreover, future studies should include measurements such as anxiety and rational or irrational cognition, which may be relevant for this situation. Future studies could also focus on different ages, since in the literature, there is a distinction in terms of the strategies used by early-identified children with learning disabilities and late-identified children with learning disabilities. 

## Figures and Tables

**Table 1:** Demographics of study participants.

Group	Gender	Urban/Rural
Typical comprehension (N = 139)	69 males	98/41 (70.5%; 29.5%)
Poor comprehension (N = 28)	17 males	17/11 (60.7%; 39.3%)

**Table 2:** Means and standard deviations of the raw scores of the Children Cognitive Assessment Questionnaire.

CCAQ Subscales	Poor Comprehension	Good Comprehension
NSE	2.39 (2.23)	1.51 (1.72)
OFFT	5.57 (2.21)	4.5 (1.79)
PSE	7.04 (4.38)	7.88 (1.99)
ONT	7.5 (1.81)	7.60 (1.88)

**Table 3:** Descriptive statistics for the raw scores of the CERQ.

CERQ Subscales	Poor Comprehension	Good Comprehension
Self-blame	9.5 (3.43)	9.86 (3.87)
Other-blame	9.68 (3.47)	7.91 (3.01)
Rumination	11.43 (3.34)	11.57 (3.93)
Catastrophizing	9.79 (3.44)	9.18 (3.74)
Putting into perspective	10.57 (3.62)	12.31 (3.71)
Positive refocusing	12.54 (3.67)	12.25 (4.37)
Positive reappraisal	10.89 (3.02)	11.70 (3.81)
Acceptance	11.04 (3.46)	11.65 (3.34)

M = mean (SD = standard deviation).

**Table 4:** Independent sample *t*-test values for the CERQ.

CERQ Subscales	*t*	Significance
Self-blame	0.48	Ns.
Other-blame *	−2.5	*p* < 0.05
Rumination	0.19	Ns.
Catastrophizing	−0.83	Ns.
Putting into perspective *	2.3	*p* < 0.05
Positive refocusing	−0.36	Ns.
Positive reappraisal	1.22	Ns.
Acceptance	0.86	Ns.
Refocus on planning	1.16	Ns. *p* = 0.081

* significant at *p* = 0.05.

## Data Availability

The data presented in this study are available on request from the corresponding author. The data are not publicly available due to restrictions related to privacy.
